# The hand grip force test as a measure of physical function in women with fibromyalgia

**DOI:** 10.1038/s41598-022-07480-1

**Published:** 2022-03-01

**Authors:** Margarita Cigarán-Méndez, Edurne Úbeda-D’Ocasar, José Luis Arias-Buría, César Fernández-de-las-Peñas, Gracia María Gallego-Sendarrubias, Juan Antonio Valera-Calero

**Affiliations:** 1 Department of Psychology, Universidad Rey Juan Carlos, Alcorcón, Madrid, Spain; 2 Department of Physiotherapy, Faculty of Health, Universidad Camilo José Cela, Calle Castillo de Alarcón 49, 28692 Villanueva de la Cañada, Madrid, Spain; 3 Department of Physical Therapy, Occupational Therapy, Rehabilitation and Physical Medicine, Universidad Rey Juan Carlos, Alcorcón, Madrid, Spain; 4 VALTRADOFI Research Group, Department of Physiotherapy, Faculty of Health, Universidad Camilo José Cela, Villanueva de la Cañada, Madrid, Spain

**Keywords:** Health care, Health occupations, Rheumatology, Risk factors, Signs and symptoms

## Abstract

Previous studies have reported the presence of muscle weakness in women with fibromyalgia syndrome (FMS) which is considered a risk factor for developing earlier disability and dependence during activities of daily life (ADL). We aimed to assess the relationship between hand grip force with sociodemographic, clinical, disease-specific, cognitive, and physical function variables in women with FMS. One hundred twenty-six women with FMS completed demographic (age, gender, height, weight, body mass index), pain-related (pain history, pain intensity at rest and during ADL), disease-specific severity (Fibromyalgia Impact Questionnaire -FIQ-S-, Fibromyalgia Health Assessment Questionnaire -FHAQ-, EuroQol-5D, Pain Catastrophizing Scale -PCS-, Pittsburgh Sleep Quality Index-PSQI-, Pain Vigilance and Awareness Questionnaire -PVAQ-, and Central Sensitization Inventory -CSI-), psychological (Tampa Scale for Kinesiophobia, TKS-11; Pain Vigilance and Awareness Questionnaire, PVAQ; Pain Catastrophizing Scale, PCS), and physical function (hand grip force, and Timed Up and Go Test, TUG). Hand grip force was associated with height (r = −0.273), BMI (r = 0.265), worst pain at rest (r = −0.228), pain during ADL (r = −0.244), TUG (r = −0.406), FHAQ (r = −0.386), EuroQol-5D (r = 0.353), CSI (r = −0.321) and PSQI (r = −0.250). The stepwise regression analysis revealed that 34.4% of hand grip force was explained by weight (6.4%), TUG (22.2%), and FHAQ (5.8%) variables. This study found that hand grip force is associated with physical function indicators, but not with fear-avoidance behaviors nor pain-related features of FMS. Hand grip force could be considered as an easy tool for identifying the risk of fall and poorer physical health status.

## Introduction

Fibromyalgia syndrome (FMS) is a chronic pain condition affecting up to the 6.6% of the general population^1^, being the third most common musculoskeletal condition after lumbar pain and osteoarthritis^2^. The annual economic burden per FMS patient is estimated at $3804^3^. FMS is characterized by a plethora of symptoms including widespread pain, physical and mental fatigue, morning stiffness, anxiety, depression, cognitive dysfunctions, sleep problems, autonomic disturbances, exacerbated pain responses (indicators of central sensitization), and reduced health-related quality of quality of life (QoL)^4–6^.

Consistent evidence identifies several sociodemographic, physical, biological, lifestyle, and psychological factors, such as depression, obesity, older age, dietary patterns, smoking, alcohol consumption, higher cortisol levels, greater risk of fall, female sex, lower income and education level, associated with frailty affecting health-related QoL and activities of daily life (ADL)^7^. Therefore, the fact that the age peak for suffering from FMS is 50–60 years old, the presence of altered cortisol levels, the high prevalence of other associated comorbidities (e.g., anxiety and depression), and considering that 80–90% of FMS sufferers are women^8,9^ could explain the high impact of FMS on QoL and ADL.

In addition, muscle weakness is a risk factor for developing earlier disability onset and dependence during ADL^10^ and a preventable contributor to the global burden of morbidity and mortality^11^. Previous reports have shown that muscle strength is generalized reduced up to 35% of magnitude when comparing FMS women with healthy women^12^. This generalized weakness may be explained by pathologic changes in muscle fibers, impaired blood circulation, disturbances in regulation of growth and energy metabolism, altered neuromuscular control mechanisms associated with pain and decreased levels of physical activity associated with fear avoidance behaviors, pain, and overweight^13,14^.

The Hand Grip Force is a reliable, simple, and noninvasive test assessing the strength of the hand muscles used to grasp or grip^15,16^. This test is considered as a valid indicator to identify frailty and risk of disability among elderly people as is associated with cardiovascular, respiratory, and cancer outcomes and also with mortality^17^. Evidence supports the presence of reduced hand grip force in women with FMS^18–23^. In addition, reduced hand grip force has been associated with worse severity^19,20^, higher related-disability^21^, lower pulmonary muscle strength^22^, and worse health-related quality of life^23^ in women with FMS. On the contrary, others did not find an association between hand grip force and severity^18,23^.

**Figure 1:**
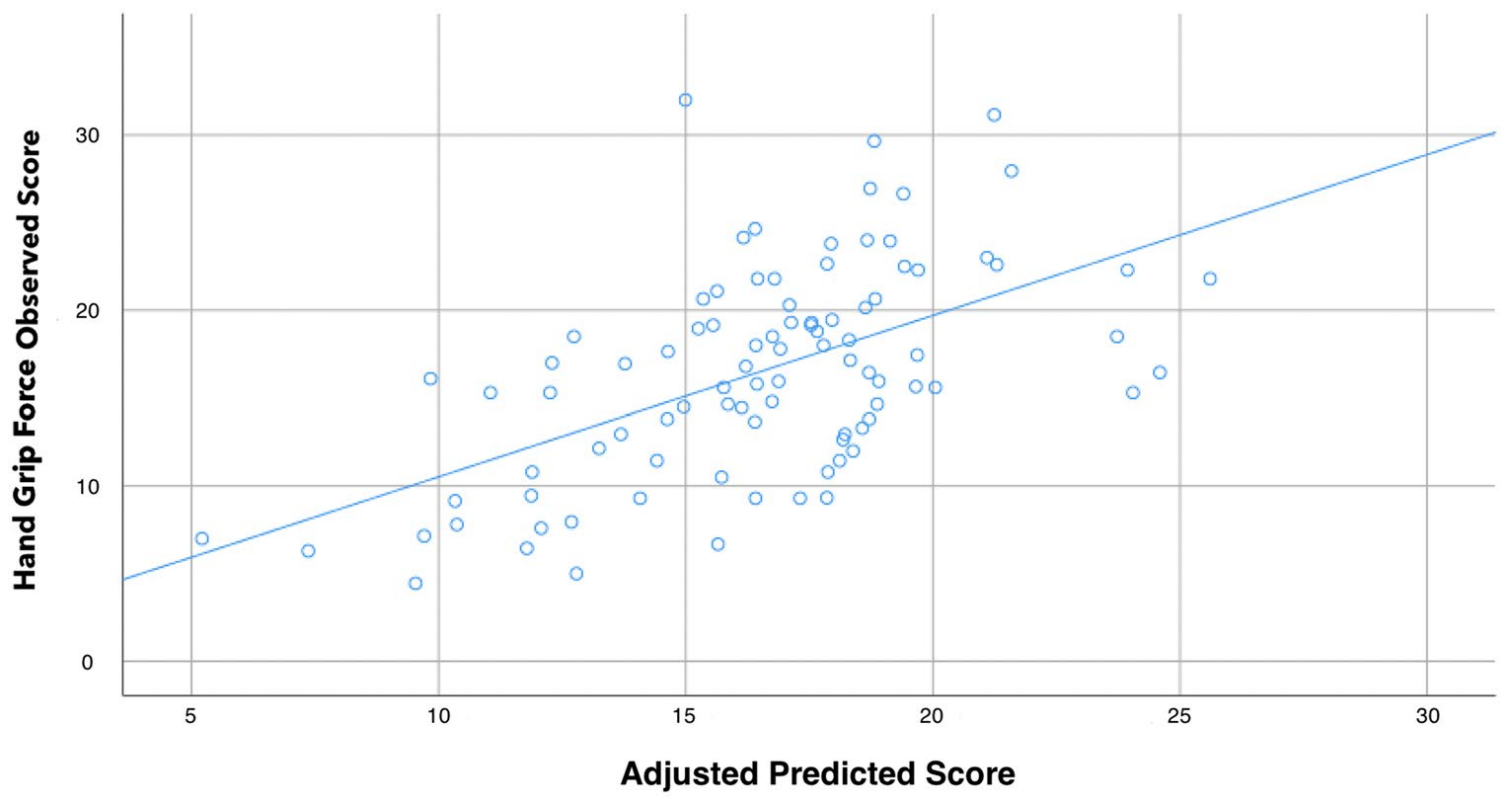
Scatter plot of the adjusted predicted score (r^2^ adjusted: 0.344) explaining Hand Grip Force score in female patients with fibromyalgia syndrome.

The Time Up and Go test is a physical performance test complementary to the hand grip commonly used tool for screening falls risk and is also considered as a strong predictor of short-term mortality^24,25^. Most previous studies conducted in FMS included sample sizes < 50 participants and did not consider other variables such as catastrophizing or kinesiophobia which could affect physical function. In fact, an association between physical capacity, including hand grip force, with fear avoidance beliefs has been suggested in FMS^13^ but also in other pain conditions, e.g., low back pain^26^. Therefore, the aims of the current study were to analyze the correlation between demographic, pain-related, disease-specific, psychological, physical function measures and hand grip force and also to develop a linear regression model to analyze factors explaining the variance of hand grip force in women with FMS (Fig. 1).

## Methods

### Study design

An observational cross-sectional study following the Strengthening the Reporting of Observational studies in Epidemiology (STROBE) guidelines^27^ was conducted. The study was approved by the Institutional Ethics Committee of Camilo José Cela University (UCJC 20-10-2020) and Universidad Rey Juan Carlos (URJC 08-30-2020). All participants signed written informed consent prior to their inclusion in the study.

### Participants

Consecutive women diagnosed of FMS^28^ by a rheumatologist and aged between 20 and 70 years old who voluntary responded to a local announcement at AFINSYFACRO Fibromyalgia Association in Madrid (Spain) were screened for eligible criteria. Exclusion criteria included: (1) previous history of whiplash injury, (2) previous surgery; (3) neuropathic condition (e.g., radiculopathy or myelopathy) diagnosed; (4) underlying medical condition (e.g., tumor); or, (5) regular use of drug pharmacological treatment affecting muscle tone or pain perception different than analgesics.

### Hand grip force

To evaluate the maximal voluntary hand grip force contraction, a Jamar hand dynamometer (JLW Instruments, Chicago, IL, USA) was used. The Jamar Hydraulic Hand Dynamometer is an enclosed hydraulic system recording the applied strength in kilograms^16^. The examiner explained and demonstrated the procedure before data collection. To perform the measurement, each subject placed the Jamar in their hand, with the arm beside the trunk, the shoulder in a neutral position, the elbow flexed at 90° and pulled the metal bar with their fingers^29^. This procedure was conducted bilaterally. The mean of three trials for each side (with 3 min resting periods between repetitions to avoid fatigue) was calculated^29^. The handle diameter was set at the 19.7% of the participant’s hand length (calculated from the crease of the wrist to the tip of the middle finger with the hand while holding straight and stiff) as recommended by Kong et al.^30^. The reliability of hand grip force in FMS has been shown to be excellent^31^.

### Pain-related and disease-specific variables

Participants were asked to rate in a 11-point numerical point rate scale (NPRS) their mean pain intensity at rest, the worst pain at rest, and the mean level of pain experienced during ADL. In this scale, 0 was interpreted as “complete absence of pain” and 10 as “the worst imaginable pain”^32^.

The Spanish version of the fibromyalgia impact questionnaire (FIQ-S) was used to assess the impact of FMS^32^. This self-reported questionnaire consists of 10 subscales assessing the daily-tasks function, the number of days feeling good during the last 7 days, the interference of FMS with their work, pain intensity, fatigue, night resting, stiffness, anxiety, and depression. The final score ranges from 0 to 100, where greater scores involve greater disability and severity and its reliability has been shown to be good reliability (ICC: 0.81)^33^.

The Fibromyalgia Health Assessment Questionnaire (FHAQ) is a disease-specific tool used for assessing functional ability in FMS^34^. Although this self-questionnaire come from the Health Assessment Questionnaire, the FHAQ is a shorter, simpler and easier to quantify in a single questionnaire with 8 items with scores ranging from 0 to 3^34^. The FHAQ final score is calculated as the mean of the 8 items, where lower scores (0) mean less difficulty during their daily functional activities.

Health-related quality of life was assessed with the paper-based five-level version of EuroQol-5D questionnaire since it can be used in specific clinical populations. The EuroQol-5D includes five descriptive health dimensions (mobility, self-care, daily activities, pain and depression/anxiety) ranging from 1 (no problems) to 3 (severe problems). The combination of the scores results in a five-digit value with 243 combinations^35^. Responses were converted into a single index number between 0 and 1 where 0 corresponds to a health state judged to be equivalent to death and 1 corresponds to optimal health, by applying crosswalk index values for Spain life^36^.

Sensitization associated-symptoms were assessed with the Central Sensitization Inventory (CSI) since it has excellent test–retest reliability and internal consistency^37^. This self-reported questionnaire contains a 25-items survey assessing the frequency of symptoms associated with sensitization, rating each question on a 5-point Likert scale where 0 means “never” and 4 means “always”. A total score ranging from 0 to 100 is obtained where a higher total score is associated with more sensitization-related symptoms^37^.

The Spanish version of the Pittsburgh Sleeping Quality index (PSQI) was used to determine sleep quality^38^. This valid and reliable self-reported questionnaire consists of 19 questions measuring the subjective sleep quality, duration and latency of sleep, sleep disorders, habitual sleep efficiency, daytime dysfunction and sleep medication in a 0 to 3 scale. The score ranges from 0 (the best sleep quality) to 21 (the worst sleep quality)^38^. The PSQI has shown proper psychometric properties for assessing sleep quality in FMS^39^.

### Psychological variables

The 11-item short-form of the Tampa Scale for Kinesiophobia (TKS-11)^40^ was used to quantify the fear of movement since its use is indicated in chronic pain conditions including FMS^41^. This self-reported questionnaire consists of 11 items where patients have to choose in a 4-point Likert scale how much they agree with each item, being 1 “complete disagreement” and 4 “complete agreement” (total score from 0 to 44)^40^.

Pain hypervigilance was assessed with the short-form 9-item Spanish Pain Vigilance and Awareness Questionnaire (PVAQ) since this self-reported questionnaire is considered a valid and reliable tool to identify ideas of observing, monitoring and focusing on pain in FMS^42^.

The Spanish version of the Pain Catastrophizing Scale (PCS) is a 13-item self-reported questionnaire used to assess the patient’s pain catastrophizing response which has demonstrated to be valid for being used in patients with FMS^43^. All items are answered in a 5-point Likert scale where 0 means “never” and 4 means “always” (score 0–52). This questionnaire analyzes three scales: rumination (constant worry and inability to inhibit thoughts related to pain), magnification (exaggeration of unpleasantness of painful situations and expectations of negative consequences) and despair (inability to face pain)^43^.

### Timed up and go test

We used the Timed Up and Go (TUG) test since is considered an easy, cheap, fast and valid tool providing valuable predictive information to identify patients at a risk of falls^25^. The patient is placed in sitting position in an armchair and is asked to stand up without the use of the arms, to walk at a comfortable and safe speed up to a line placed at 3 m from the chair, to turn back to the chair, and sit down again. The TUG has shown to be a reliable physical fitness test for assessing agility/dynamic balance in women with FMS^44^.

### Sample size calculation

Sample size calculation was estimated using the G*Power software for Mac OS (v.3.1.6) based on detecting significant small correlations (r = 0.15) between the variables with an alpha level (α) of 0.05 and a desired power (β) of 90%. This generated a sample size of at least 108 participants.

### Statistical analysis

The Statistical Package for Social Sciences (SPSS) software v.25 for Mac OS (IBM, Armonk, NY) was used for all statistical analyses. A descriptive analysis was used to describe the sample central tendency and dispersion of all variables. Normal distribution of quantitative variables was verified with Kolmogorov–Smirnov test. Since no side-to-side differences in hand grip force were seen (student t-test for independent samples), the mean was used in the analyses.

The potential associations between the variables were calculated in a correlation matrix by calculating Pearson correlation coefficients (r). In addition, r scores were used to identify multicollinearity and shared variance between the variables (defined as r > 0.80). All statistically significant variables associated with hand grip force (dependent variable) were included into a stepwise multiple linear regression model to quantify their final contribution. The significance criterion of the critical F value for entry into the regression equation was set at *P* < 0.05. The following independent variables were considered: age, height, weight, body mass index, years with pain, years with FMS, pain intensity, TUG, FIQ-S, FHAQ, EuroQol-5D, CSI, PVAQ, PCS, TSK-11 and PSQI. Changes in adjusted *R*^2^ were reported after each step of the regression model to determine the association of the additional variables.

### Ethical approval

The Local Ethics Committee of Camilo José Cela University (UCJC 20–10-2020) and Universidad Rey Juan Carlos (URJC 08–30-2020) approved the study design.

### Informed consent

Informed consent was obtained from all subjects involved in the study.

## Results

One-hundred and forty (*n* = 140) women with FMS responded to the announcement and were screened for eligibility criteria. Fourteen (10%) were excluded due to previous surgery (*n* = 8), previous whiplash (*n* = 4), and pregnancy (*n* = 2). After exclusion filtering, 126 women (mean age: 52.2 ± 10.7 years) were included. Table 1 shows data of the total sample regarding demographic, pain-related, disease-specific, psychological and physical function.

**Table 1:** Baseline outcomes (mean ± SD) of the sample.

Baseline variable	Female patients with FMS (*n* = 126)
**Sociodemographic characteristics**	
Age (years)	52.0 ± 10.7
Height (m)	1.61 ± 0.06
Weight (kg)	71.4 ± 16.6
Body mass index (kg/cm^2^)	27.5 ± 6.2
**Clinical characteristics**	
Years of pain (years)	20.1 ± 15.3
Years with FMS (years)	10.2 ± 8.9
Mean pain at rest (0–10)	6.4 ± 1.7
Worst pain at rest (0–10)	7.3 ± 2.2
Pain during daily activities (0–10)	8.1 ± 1.9
Time up and go test (seconds)	12.4 ± 4.9
Fibromyalgia impact questionnaire (0–100)	64.8 ± 12.7
Fibromyalgia health assessment questionnaire (0–3)	1.3 ± 0.6
EuroQol-5D questionnaire (0–100)	0.41 ± 0.25
Central sensitization inventory (0–100)	70.7 ± 11.6
Pain vigilance and awareness questionnaire (0–45)	27.0 ± 8.2
Pain catastrophizing scale (0–52)	22.5 ± 12.3
Tampa scale for kinesiophobia TSK-11 (0–44)	24.9 ± 7.5
Pittsburgh sleeping quality index (0–21)	13.8 ± 3.9
Hand grip force (kg)*	
Mean	16.7 ± 6.2
Non-dominant hand	16.5 ± 6.4
Dominant hand	17.1 ± 6.4

*No side-to-side differences (*p* = 0.494).

### Bivariate correlation analysis

Bivariate correlation analysis results are reported in Table 2. Hand grip force was negatively associated with the worst pain, PSQI (both, *p* < 0.05), mean pain during ADL, TUG, FHAQ and CSI (*p* < 0.01) and positively associated with BMI (*p* < 0.05), height and EuroQol-5D (*p* < 0.01). Thus, multiple significant correlations existed among different outcomes (r: 0.189 to 0.640), but none fulfilled multicollinearity level (all, r < 0.80).

**Table 2:** Pearson-product moment correlation matrix between sociodemographic, physical and clinical characteristics.

	1	2	3	4	5	6	7	8	9
1. Age									
2. Weight	n-s								
3. Height	-.190*	.278**							
4. BMI	n-s	.948**	n-s						
5. Years with pain	.566*	n-s	-.220*	n-s					
6. Years with FMS	.598**	n-s	n-s	n-s	.615**				
7. Mean pain at rest	n-s	n-s	n-s	n-s	n-s	n-s			
8. Worst pain at rest	n-s	n-s	n-s	n-s	n-s	n-s	.427**		
9. PADL	n-s	n-s	n-s	n-s	n-s	n-s	.302**	n-s	
10. TUG	.190*	.233**	n-s	.251**	n-s	n-s	n-s	n-s	.297**
11. FIQ	-.199*	n-s	n-s	n-s	n-s	n-s	.421**	.272**	.422*
12. FHAQ	n-s	.178*	n-s	.202*	n-s	n-s	.251**	n-s	.466**
13. EQ5D	n-s	n-s	n-s	n-s	n-s	n-s	-.320**	-.217*	-.396**
14. CSI	-.262**	n-s	n-s	n-s	n-s	n-s	.305**	.249**	.398**
15. PVAQ	n-s	n-s	n-s	n-s	n-s	n-s	.316**	.234*	.210*
16. PCS	n-s	n-s	n-s	n-s	n-s	n-s	.258**	.184*	.385**
17. TSK11	n-s	.244**	n-s	.218*	n-s	n-s	n-s	n-s	.344**
18. PSQI	-.202*	n-s	n-s	n-s	-.189*	n-s	n-s	n-s	n-s
19. Hand Grip Force	n-s	.273**	n-s	.265*	n-s	n-s	n-s	-.228*	-.244**

	10	11	12	13	14	15	16	17	18
1. Age									
2. Weight									
3. Height									
4. BMI									
5. Years with pain									
6. Years with FMS									
7. Mean pain at rest									
8. Worst pain at rest									
9. PADL									
10. TUG									
11. FIQ	n-s								
12. FHAQ	.471**	.403**							
13. EQ5D	-.349**	-.455**	-.594**						
14. CSI	.279**	.459**	.580**	-.571**					
15. PVAQ	n-s	.298**	.187*	-.287**	.217*				
16. PCS	.233**	.388**	.395**	-.543**	.456**	.526**			
17. TSK11	.317**	.199*	.437**	-.448**	.368**	.311**	.640**		
18. PSQI	n-s	n-s	n-s	-.246**	.215*	n-s	.198*	n-s	
19. Hand Grip Force	-.406**	n-s	-.386**	.353**	-.321**	n-s	n-s	n-s	-.250*

BMI, Body Mass Index; CSI, Central Sensitization Inventory; EQ5D, EuroQol-5D Questionnaire; FHAQ, Fibromyalgia Health Assessment Questionnaire; FIQ, Fibromyalgia Impact Questionnaire; FMS, Fibromyalgia syndrome; PCS, Pain Catastrophizing Scale; PDDA, Pain during activities of daily life; PSQI, Pittsburgh Sleeping Quality Index; PVAQ, Pain Vigilance and Awareness Questionnaire; TSK-11, Tampa Scale for Kinesiophobia; TUG, Time Up and Go test.

*P < 0.05; **P < 0.01.

### Multiple regression analysis

The hierarchical regression analysis explaining the variance of hand grip force is shown within Table 3. Stepwise regression analyses revealed that weight (contributing 6.4%), TUG (additional 22.2%), and FHAQ (additional 5.8%) were significant contributors for hand grip force and, when combined, they explained 34.4% of the variance (r^2^ adjusted: 0.344). Variance should be interpreted as a measure of how far the observed values differ from the average of predicted values and it ranges from 0 (the dependent variable cannot be explained based on the independent variables listed) to 1 (the dependent variable can be totally explained based on the independent variables).

**Table 3:** Summary of the stepwise regression analyses to determine predictors of hand grip force.

	Predictor outcome	Β	SE B	95% CI	β	t	*P*
Hand grip force	Step 1						
	Weight	.109	.040	.029; .188	.273	2.696	.029
	Step 2						
	Weight	.147	.036	.076; .219	.371	4.102	< .001
	Time up and go	-.672	.125	-.920; -.424	-.486	-5.380	< .001
	Step 3						
	Weight	.147	.034	.079; .215	.370	4.272	< .001
	Time up and go	-.513	.131	-.774; -.253	-.372	-3.916	< .001
	FHAQ	-3.062	1.030	-5.109; -1.015	-.277	-2.973	.004

FHAQ, Fibromyalgia Health Assessment Questionnaire.

R^2^ adj. = .064 for step 1, R^2^ adj. = .286 for step 2, R^2^ adj. = .344 for step 3.

## Discussion

This is the first study performing a regression analysis to identify potential contributors explaining the variance of hand grip force in a female sample of women with FMS. Hand grip force is a reliable and easy to administer way to measure overall physical capacity and muscle strength and a good predictor for impairment, disability and functional ability in a wide variety of rheumatic and neurological conditions^45^. In fact, recent evidence suggests hand grip force as a possible biomarker of impaired neuromuscular function and a predictor of risk of falls in older women^46^. Therefore, understanding hand grip force associated factors is essential to develop early screening tools and treatment programs for specific targets in order to avoid the development of earlier disability onset, dependence during ADL, impaired quality of life and early mortality caused by possible accidental falls^10–14,19–23^. This study revealed that hand grip force was associated with taller size, greater BMI, lower pain intensity at rest and during ADL, better quality of life (i.e., FHAQ and EQ5D), lower sensitization-associated symptoms (i.e., CSI), TUG, and poor sleep quality (i.e., PSQI). These findings support the importance of focusing on physical function assessment and strengthening by including exercise programs as they are clinically effective, no adverse effects and cost-effective complement to the usual care^47^.

Previous evidence suggests an impact of sex on hand grip force. As hand strength and hand length are closely associated and the fact that hand length is significantly greater in men, this may explain differences^48^. Similarly, although we did not include male participants due to its limited prevalence and, accordingly, sex differences could not be assessed, we found that demographic features were associated with hand grip force. As hand length can also vary within the same gender as is also associated with anthropometry characteristics, this may explain the height and BMI association with hand grip force. Further, sex differences are more pronounced for the non-dominant than for the dominant hand^48^. Our results showed that anthropometric variations within the same sex were not enough relevant to involve strength differences between dominant and non-dominant hands.

Pain is the most common symptom associated with FMS and was also associated with hand grip force in the current study. These findings reinforce previous evidence explaining the association between pain intensity with a delay on hand grip initiation, release, and force^49^. Two mechanisms could explain an initiation delay and prolonged release timings: the avoidance of muscle contraction as a protective mechanism against pain and attributable to fear-avoidance behaviors or hypervigilance, and a reduced quality of sensory feedback caused by sensitization^49^. A decreased muscle contraction capacity, fear of pain and reinjury and histological muscle changes could explain the decrease muscle strength^50^. Interestingly, even if hypervigilance, kinesiophobia or catastrophism were associated with multiple variables (pain intensity, quality of life, fibromyalgia impact, sensitization and sleep quality), no association with hand grip force was observed. Other variables (e.g., quantitative sensory tests, neuropathic pain component and psychological factors) not included in our study should be assessed in further research.

Thus, we analyzed the association between hand grip force with life of quality indicators, sleep quality, fibromyalgia impact and TUG. Hip fractures derived from falls are associated with up to a 29% of mortality within one year after injury^51^. As muscle weakness is the most common modifiable risk factor (in addition to balance deficits and gait instability) associated with fall^52^ and considering the observed association between FMS impact with TUG, quality of life, kinesiophobia, catastrophism and pain hypervigilance, muscular fitness testing should be included in clinical practice and multidisciplinary exercise programs aiming at improving physical function would be essential to reduce disability, dependence, and risk of falls.

Finally, some potential limitations should be recognized. First, this study consisted of a female sample as male participants accessibility was limited. Therefore, our findings cannot be extrapolated to males. Second, we did not assess pain feature (e.g., neuropathic component), quantitative sensory testing or psychological aspects (e.g., anxiety and depression), which could be potentially associated with physical function in chronic widespread pain syndromes. Finally, longitudinal studies to confirm the association between hand grip force and risk of falls in patients with FMS are needed.

## Conclusions

This study found hand grip weakness to be associated with greater sensitization and pain intensity (at rest and during ADL) and poorer quality of life, functional ability and sleep quality whereas larger TUG scores were associated with greater sensitization and poorer quality of life, functional ability, pain catastrophism and kinesiophobia. These finding suggest that both physical tests assess different aspects of FMS and provide synergistic information. Therefore, muscular fitness could be a complementary screening tool during clinical practice and aiming the physical condition strengthening could induce a positive impact on patients’ quality of life, independence, delay on disability onset and risk of early mortality caused by accidental falls.

## Data Availability

All data derived from this study are presented in the text.
